# Circular RNAs in osteoporosis: expression, functions and roles

**DOI:** 10.1038/s41420-021-00624-8

**Published:** 2021-09-04

**Authors:** Yinzhou Luo, Guanzhen Qiu, Yize Liu, Shanshan Li, Yeqiu Xu, Yuanzhuang Zhang, Yuan Cao, Yong Wang

**Affiliations:** 1 Fourth Department of Orthopedic Surgery, Central Hospital Affiliated to Shenyang Medical College, 110024 Shenyang, Liaoning P.R. China; 2 Department of Respiratory, Central Hospital Affiliated to Shenyang Medical College, 110024 Shenyang, Liaoning P.R. China; 3 Central Laboratory, Central Hospital Affiliated to Shenyang Medical College, 110024 Shenyang, Liaoning P.R. China

**Keywords:** Long non-coding RNAs, Non-coding RNAs

## Abstract

Osteoporosis, which is caused by an imbalance in osteoblasts and osteoclasts, is a global age-related metabolic disease. Osteoblasts induce osteocyte and bone matrix formation, while osteoclasts play an important role in bone resorption. Maintaining a balance between osteoblast formation and osteoclastic absorption is crucial for bone remodeling. Circular RNAs (circRNAs), which are characterized by closed-loop structures, are a class of novel endogenous transcripts with limited protein-coding abilities. Accumulating evidence indicates that circRNAs play important roles in various bone diseases, such as osteosarcoma, osteoarthritis, osteonecrosis, and osteoporosis. Recent studies have shown that circRNAs regulate osteoblast and osteoclast differentiation and may be potential biomarkers for osteoporosis. In the current review, we summarize the expression, function, and working mechanisms of circRNAs involved in osteoblasts, osteoclast differentiation, and osteoporosis.

## Facts

- CircRNAs, a great type of ncRNAs which participated in adjusting genetic transcription and translation, are verified to play vital roles in the regulation of bone diseases, such as osteosarcoma and osteoporosis.
- The imbalance in osteoblast and osteoclast gives rise to osteoporosis, which is characterized by decreased bone mass, microarchitectural deterioration, and fragility fractures.
- Accumulative evidences are revealing circRNAs could be regulatory factors in osteoporotic homeostasis.

## Open questions

- What is the role of regulatory circRNAs in osteoblast, osteoclast, and osteoporosis?
- What are the regulative mechanisms circRNAs leading in osteoblast and osteoclast?
- How can we aggregate circRNAs’ preclinical achievement into the clinical application?

## Introduction

Osteoporosis is a common disease that occurs in ~35% of women older than 65 years of age. When the dynamic balance in osteoblastic bone formation and osteoclastic bone resorption is broken, endocrine metabolism emerges with, combined with osteoporosis [1, 2]. Osteoporosis is characterized by decreased bone mass, microarchitectural deterioration, and fragility fractures [3]. The reduced contribution of osteoblasts to bone mass can explain why bone thickness is reduced, which characterizes osteoporosis [4]. It found that osteoporosis was caused by disturbing various target sites along the pathway of osteoblast proliferation, differentiation, and activation [5]. Knowing the molecular mechanisms that mediate osteoblast and osteoclast differentiation will provide a comprehensive understanding of osteoporosis.

CircRNAs, a novel group of noncoding transcripts, do not possess 3’ and 5’ ends but instead form a closed loop that is dissimilar to linear RNAs. CircRNAs were first detected in a virus by Sanger in 1976 and were deemed irrelevant byproducts without any significant biological functions for a period of time. Later, as emerging studies on noncoding RNAs were performed and new technologies were developed, thousands of circRNAs were discovered. Currently, the biogenesis of different circRNAs has been discovered. CircRNAs have been described with universal characteristics such as stability and conservation, and a majority of circRNAs could be biomarkers in clinical practice and research [6]. CircRNAs regulate the processes of various disease pathologies, including osteosarcoma, osteoarthritis, lumbar intervertebral disc degeneration, and osteoporosis [2, 7–9]. In osteoporosis, circRNAs participate in proliferation, differentiation, and apoptosis. Several reviews have described the classic expression and functions of circRNAs and linear RNAs, and the mechanisms and new expression are summarized in the present review.

## Biogenesis and characteristic of circRNAs

CircRNAs, which are covalently closed circular RNAs produced from exons and introns of genes in eukaryotes, are often generated by backsplicing of the corresponding precursor mRNA [10]. CircRNAs are often expressed at low levels and are generally cell-specific and tissue-specific.

**Fig. 1:**
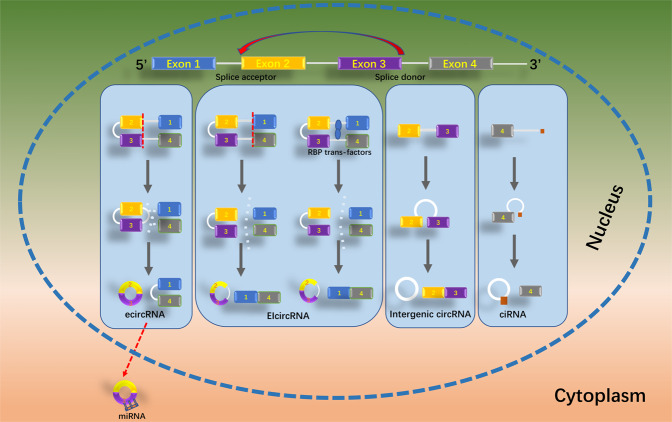
Schematic diagram of classifications of circRNAs. According to the biogenesis characteristics from genomic regions, circRNAs are often divided into four types, ecircRNA, EIcircRNA, intergenic circRNAs, and ciRNA. ecircRNA exon circRNAs, EIcircRNA exon–intron circRNAs, ciRNA circular intronic RNA.

According to diverse biogenesis characteristics from genomic regions, circRNAs are often divided into four types: intron circRNAs, exon circRNAs, intergenic circRNAs, and exon–intron circRNAs (EIciRNAs) [11]. The majority of circRNAs contain 3’ → 5′-linked exon sequences [12], which are called exon circRNAs. Intron circRNAs include circular intron RNAs and excised group I/II/tRNA introns [13]. EIciRNAs are often nuclear circRNAs, which are formed by exons and introns [10]. Intergenic circRNAs are nonexon circRNAs [11] (Fig. 1).

CircRNAs are a type of transcript with high conservation and stability, enabling circRNAs to be meaningful biomarkers in the clinic. The biogenesis of circRNAs in the nucleus and cytoplasm involves up to three main mechanisms, including core spliceosomal machinery, *cis* elements, and RNA-binding proteins (RBPs) [10]. By depleting core spliceosomal components, such as the SF3a and SF3b complexes, the expression of circRNA was preserved [14]. The upstream 3’ ss is reversely ligated with the downstream 5’ ss, and the core spliceosomal machinery induces a low-efficiency process. Backsplicing, which is the only method of circRNA formation, requires regulatory parts within introns flanking circularized exons.

Due to the high level of stability of circRNAs, it is easy to detect circRNAs noninvasively in body fluids [15]; thus, the majority of studies have focused on the expression of circRNAs in solid tumors. To date, different functions of circRNAs have been examined in various tissues and cells.

### Circular RNAs regulate splicing and transcription

In *Zea mays*, centromeric retrotransposons transcribe backsplicing; as a result, circRNAs bind to centromeres and increase the level of chromatin looping with an R-loop structure [16]. This is a novel angle from which to study how this mechanism develops in the nucleus.

### Circular RNAs can act as microRNA sponges

CircRNAs can work as ceRNAs to bind miRNAs and prevent them from inhibiting their target mRNAs. As a classic circRNA, CDR1as, which contains 70 binding sites for miR-7, is richly expressed in the brain [17].

### Circular RNAs work as protein scaffolds

By interacting with U1 small nuclear ribonucleoproteins, exon–intron-containing circRNAs promote parent genes at the transcript level [12]. CircFoxo3 is associated with cell cycle progression. Cyclin-dependent kinase 2 (CDK2) and cyclin-dependent kinase inhibitor 1 (p21) can bind to circFoxo3 and repress CDK2 functions [18].

### CircRNAs can be translated

Internal ribosome entry sites (IRESs) promote circRNA translation by promoting the binding of initiation factors or ribosomes to circRNAs [19]. It has been shown that some endogenous circRNAs can be translated, and circFBXW7 can be translated into the 21-kDa protein FBXW7 [20]. Based on new evidence, the translation of circRNAs is reduced by m6A demethylase fat mass and obesity-associated protein but increased by METTL3/14 [21], elucidating a new mechanism of circRNA translation.

Large numbers of circRNAs have been shown to play crucial roles in osteoporosis. For example, by studying 40 women with PMO (postmenopausal osteoporosis), Liu et al. discovered that circZNF720 (circbase ID: circ_0007059) could attenuate osteoclast differentiation through the microRNA-378/BMP2 axis, which in turn repressed osteoporosis [22]. circPRIM2 (circbase ID: hsa_circ_0076906) binds to miR-1305 to regulate the expression of OGN (osteoglycin) and alleviate osteoporosis [23]. During this process, osteogenic differentiation was a main regulatory factor of circPRIM2 via the miR-1305/OGN pathway. In conclusion, circRNAs play important roles in the regulation of osteoporosis and the processes of osteoblastic differentiation and osteoclastic differentiation. However, the detailed mechanisms of these processes remain to be elucidated. In the present review, we summarize the expression, function, and working mechanisms of relevant circRNAs in osteoporosis, osteogenesis, and osteoclastogenesis.

## Aberrant expression of circRNAs in osteoporosis

Similar to other diseases, a large number of circRNAs are differentially expressed in osteoporosis. The upregulation and downregulation of circRNAs were shown to participate in the pathological processes of osteoporosis, accelerating or repressing osteoporosis (Table 1). Three samples with different levels were collected, and the laboratory results are described.

**Table 1:** The dysregulated circRNAs in osteoporosis.

Gene symbol (circBase ID)	Function on osteoporosis	Expression	Downstream genes	PMID
CDR1as (hsa_circ_0001946)	Inhibition	Upregulated during the osteogenic differentiation	miR-7/GDF5	30170617
CDK8 (hsa_circ_0003489)	Represses the osteogenic differentiation of PDLSCs	Increased in periodontitis tissues	/	32978798
SIPA1L1 (hsa_circ_0102538)	Promote osteogenic differentiation	Down expressed in SCAPs during the osteogenic differentiation	miR-204-5p/ALPL	33138854
Hmbox1 (hsa_circ_0083821)	Inhibit RANKL-induced osteoclasts differentiation	Reduced in TNF-α-induced osteoclast formation	miR-1247-5p/Bcl6	33425899
CSNK1G3 (hsa_circ_0127780)	Promotion	Upregulation	miR‐335/ Dkk‐1	30046991
YAP1 (hsa_circ_0024093)	Facilitate osteoblast differentiation in BMSC and MC3T3-E1 cells	Increased during differentiation of BMSC and MC3T3-E1 cells	miR-376b-3p/YAP1	32768931
circ_28313	Promotion	Upregulation	/	31204558
Dbf4 (mmu_circ_0001314)	Promote osteoclastogenesis	Increased during osteoclast differentiation	miR-5107/ TRAF6	32744684
ZNF720 (mmu_circ_0007059)	Circ_0007059 overexpression attenuated hBMSC differentiation into osteoclasts in vitro	Upregulated in patients with PM	miR-378/BMP2	33200464
circ_33287	Promote the osteogenic differentiation of MSMSCS	Upregulated during the osteogenic differentiation of MSMSCS	/	30551425
DNAH14 (hsa_circ_0016624)	Prevent osteoporosis	Downregulated in osteoporosis	miR-98/BMP2	31235259
MIDN (hsa_circ_0048211)	Alleviate the progression of PMOP	Downregulated in hBMSCs isolated from PMOP patients	miR-93-5p/BMP2	32329818
RUNX2 (hsa_circ_0076690)	Promote osteogenic differentiation	Decreased in osteoporosis patients	miR152/RUNX2	32717724
ZCCHC17 (hsa_circ_0011269)	Inhibit osteoporosis	Increased during osteogenic differentiation	miR-122/RUNX2	32619044
DYNC1H1 (hsa_circ_0002060)	Could be used as a potential diagnostic biomarker and therapeutic target in osteoporosis	Upregulated in osteoporosis patients	/	31056800
PRIM2 (hsa_circ_0076906)	Relieved osteoporosis	Circ_0076906 silencing inhibited osteogenesis-related genes in hMSCs.	miR-1305/OGN	32087327
PLCL2 (hsa_circ_0001275)	Potential diagnostic biomarker for PMOP	Upregulated in PMOP patients	/	29742503
circ_SLC8A1	Inhibition	Reduced in osteoporosis	miR-516b-5p/AKAP2	32830397
SLC4A7 (hsa_circ_0006215)	Overexpression of hsa_circ_0006215 promotes the osteogenic differentiation of BMSCs.	Downregulated in BMSCs from patients with OP	miR-942-5p/RUNX2, VEGF	33819188
SMARCA5 (hsa_circ_0001445)	A novel biomarker for osteoporosis	Much lower in OPO patients compared with healthy controls	/	33140658
RPL41 (hsa_circ_0026827)	Promote osteoblast differentiation of DPSCs	Increased during osteoblast differentiation	miR-188-3p/Beclin1, RUNX1	32671065
ENC1 (hsa_circ_0006859)	Suppress osteoblastic differentiation	Upregulated in osteoporosis patients	miR-431-5p/ROCK1	33648601
Tssc1 (mmu_circ_0003865)	Inhibit osteoporosis	Decreased by MEL treatment	miR-3653-3p/ GAS1	33632317
CD44 (hsa_circ_0021739)	Inhibited the differentiation of osteoclasts	Downregulated in patients with postmenopausal osteoporosis	miR-502-5p	33875631
REV1 (hsa_circ_0001052)			miR-124-3p	33884524

The main functions and expressions of circRNAs in osteoporosis are listed in this table. Besides, we collect and list downstream genes according to the mechanisms authors studied in articles.

Through clinical research, Yu and Liu collected six serum or plasma samples from patients with osteoporosis and paired serum or plasma samples from healthy patients. A total of 387 circRNAs were differentially expressed in osteoporosis (filtration criteria: |foldchange | > 2, *P* value <0.01) compared with healthy controls. Among them, 211 circRNAs were upregulated (screened out as expressing the top 1 circRNAs (circ_0016624) from all expressed profiles), while 176 circRNAs were downregulated. The researchers showed that circDNAH14 (circBase ID hsa_circ_0016624) prevented osteoporosis through the regulation of BMP2 via miR-98 sponging [24].

Among 40 women with PMO (postmenopausal osteoporosis) and paired normal controls, Liu et al. selected five samples for RNA sequencing, which showed that 250 differentially expressed circRNAs were present in osteoporosis, 64 circRNAs showed a decreasing expression trend, and 186 other circRNAs were increased (filtration criteria: |fold alternation | > 2 and *P* < 0.01). Among the six top circRNAs with different expression levels (circ_0043813, circ_0001649, and circ_0005654 were upregulated, circ_0007059, circ_0001204, and circ_0001795 were downregulated), Liu et al. chose to examine circ_0007059 in osteoporotic samples and discovered that circ_0007059 expression was reduced in osteoporotic samples [22].

In the Zhang and Jia [25] study, almost 3938 upregulated circRNAs and 1505 downregulated circRNAs were shown to be involved in osteoblast differentiation. To verify the function of circRNA_0048211 in osteoporosis, Qiao et al. collected 60 bone marrow samples from PMOP patients and healthy patients, and these samples were cultured in osteogenic induction medium. The researchers found that circRNA_0048211 protected against PMOP by sponging miRNA-93-5p to regulate BMP2 [26].

Using postmenopausal mouse models, Wang et al. concentrated on the differentially expressed circRNAs in osteoporotic mice. The researchers used ten ovariectomized mouse models, total RNA was isolated from the mouse models, and the differentially expressed circRNAs were analyzed. In total, 387 differentially expressed circRNAs (211 upregulated circRNAs and 176 downregulated circRNAs) were found in osteoporotic mice [27].

## CircRNAs regulate osteogenesis

**Fig. 2:**
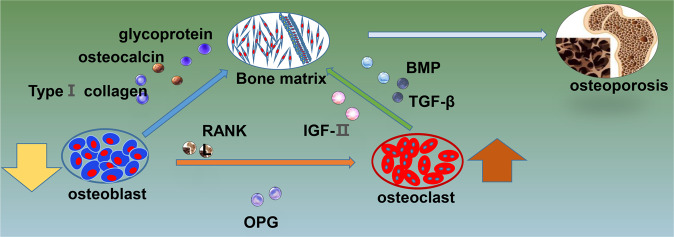
Molecular mechanism of the pathological process of osteoporosis. The pathological progress of osteoporosis is due to the imbalance between bone regeneration and bone resorption mediated by osteoblasts and osteoclasts. OPG osteoprotegerin, BMP bone morphogenetic protein, TGF-β transforming growth factor-β, RANK receptor activator of nuclear factor-κ B, IGF-II insulin-like growth factor II.

Osteoporosis could be attributed to dysregulated bone remodeling. Osteoblasts and osteoclasts share a balance between bone regeneration and bone resorption (Fig. 2). Osteogenesis, which is induced by osteoblastic differentiation, is a basic bone formation and bone remodeling process. Bone-inductive extracts of demineralized bone known as BMPs (bone morphogenetic proteins) originate from the TGF-β superfamily and play an important role in osteogenesis. BMP2 induces the formation of bone in vivo. Liu et al. showed that osteogenesis could be accelerated and relieve osteoporosis by targeting BMP2 [28].

Osteogenesis is closely connected to osteoporosis, and many studies have proven this relationship. Cai et al. discovered that silencing lncRNA-ANCR could promote osteogenesis in osteoporosis [29], and Wang et al. also showed that circRNAs could promote osteogenesis in osteoporosis by upregulating FOXO1 [7].

**Fig. 3:**
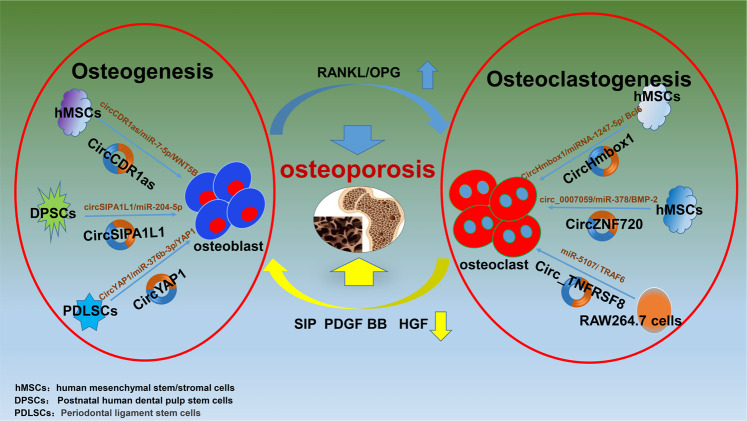
A diagram briefly summarized the circRNAs that are implicated in the dynamic balance between osteogenesis and osteoclastogenesis. PDGF platelet-derived growth factor, HGF hepatic growth factor.

Accumulating evidence has indicated that circRNAs, including CDR1as, CDK8, and SIPA1L1 (as listed in Table 1), are extensively implicated in the pathological process of osteogenesis (Fig. 3).

### Circular RNA CDR1as

Circular CDR1as arises from upstream LINC00632 and sponges miR-7 [30]. CDR1as is a common circRNA in many conditions, such as hepatocellular carcinoma, muscle development, and bladder cancer [31–33]. In a study by Li et al. [34], CDR1as was found to be upregulated during osteogenic differentiation compared with miR-7, which was downregulated in periodontal ligament stem cells. In human umbilical cord-derived mesenchymal stem cells (hucMSCs), CDR1as participates in maintaining the proliferation and differentiation of these cells [35]. However, in steroid-induced osteonecrosis of the femoral head, CDR1as plays an adverse role. Knocking down CDR1as could promote osteogenesis and decrease adipogenic differentiation in BMSCs via the CDR1as-miR-7-5p-WNT5B axis [36].

### Circular RNA CDK8

Cyclin‐dependent kinase 8 (CDK8) is a negative regulator of RNA polymerase II‐dependent transcription. A recent study identified the novel circular RNA hsa_circ_0003489, which is located at the gene region of CDK8 and is named circCDK8. This circular RNA decreases osteogenic differentiation in periodontal ligament stem cells (PDLSCs) by inducing autophagy activation via mTOR signaling [37] (Fig. 3).

### Circular RNA SIPA1L1

Signal-induced proliferation-associated 1 like 1 (SIPA1L1) is a GTPase-activating protein that is expressed downstream to regulate Rap1 activation, thus inducing gastrulation [38]. Another study focused on the circular RNA SIPA1L1, and a detailed site in the gene was not found, perhaps because it is situated on a part of the SIPA1L1 gene. In this study, circular RNA SIPA1L1 promoted osteogenic differentiation through the miR-204-5p/ALPL pathway [39]. A similar study also showed that the circular RNA SIPA1L1 could regulate osteogenic differentiation by regulating the miR-617/Smad3 axis [40] (Fig. 3).

## CircRNAs participate in osteoclastogenesis

Osteoclasts adhere to the bone matrix, originate from the monocyte/macrophage hematopoietic lineage, and secrete acid and lytic enzymes to dissolve the bone matrix. Keeping a balance between bone absorption and bone formation is necessary, and any disease may disturb this process, leading to excess osteoclastic activity. These diseases include osteoporosis, periodontal disease, and rheumatoid arthritis [41]. Bone loss is mainly induced by bone-resorbing osteoclasts, and eIF2α signaling, which plays an important role in the formation of osteoclasts, is regulated by inhibiting NFATc1 and Rac1 GTPase [42]. NFATc1, a crucial transcription factor that is a key part of the RANKL-induced signaling pathway, is repressed by cyanidin chloride. A therapeutic strategy for targeting osteoclast development in osteoporosis by inhibiting NFATc1 has been considered [43].

During osteoclastogenesis, the expression of circular RNA can differ. Dou et al. [44] showed that in mature osteoclasts, 38 circRNAs and 78 miRNAs are upregulated, and 24 miRNAs and 135 circRNAs are downregulated, which indicates that different circRNAs exhibit different trends in a given condition. Here, several crucial circular RNAs are discussed (Table 1).

### CircHmbox1

The homeobox-containing 1 (Hmbox1) gene was verified to play an important role in BMSC differentiation into vascular endothelial cells (VECs) [45]. In addition, Ma et al. [46] performed a study to examine hmbox1 in VECs and found that HMBOX1 inhibited apoptosis and promoted autophagy in VECs by regulating intracellular free zinc levels and interacting with MT2A. Lu et al. [47] also revealed that Hmbox1 participated in the differentiation of bone marrow stromal cells, and CD163 was involved in the Hmbox1/CD163/FGF-2 signaling pathway in BMSC differentiation into VECs. These studies showed that Hmbox1 could be involved in bone activities. Therefore, circHmbox1 was a novel focus in recent studies. Tumor necrosis factor-alpha (TNF-α) enhances bone resorption by promoting osteoclast differentiation and inhibiting osteoblast differentiation. Liu et al. [48] showed that the level of circHmbox1 was reduced during TNF-α-induced osteoclast formation, and circHmbox1 could inhibit RANKL-induced osteoclast differentiation, especially by binding to microRNA-1247-5p, which targets B-cell lymphoma 6 (Bcl6). This study revealed that circRNAs were implicated in TNF-α-regulated osteoclast differentiation (Fig. 3).

### Other circular RNAs in osteoclastogenesis

CircRNA_28313 knockdown greatly inhibited RANKL + CSF1-induced differentiation of osteoclasts among BMM cells in vitro but inhibited bone resorption in ovariectomized (OVX) mice in vivo [49]. circRNA_009934 (circBase ID: hsa_circ_TNFRSF8)promotes osteoclastogenesis by acting as a ceRNA of miR-5107 and upregulates the expression of TRAF6 [50] (Fig. 3). Targeting the circ_0007059/miR-378/BMP2 axis is a potential method of osteoporosis treatment, and Liu et al. indicated that circ_0007059 (circBase ID: hsa_circ_ZNF720)was upregulated in patients with PMO and during osteoclastogenesis in hBMSCs, bound to miR-378 and downregulated BMP2 expression [22] (Fig. 3).

## Functional mechanisms of circRNAs in osteoporosis

CircRNAs can function as miRNA sponges, interact with proteins, be translated into proteins and regulate transcription [10]. The main functional mechanisms of circRNAs in human osteoporosis are divided into two groups (miRNA sponges and autophagy activation), which could be involved in osteoblast and osteoclast differentiation and proliferation

### CircRNAs are implicated in osteoporosis by serving as miRNA sponges

It has already been proven that miRNAs can be complementarily paired with the UTR of mRNAs. circRNAs with MREs (miRNA response elements) can competitively bind with miRNAs, which have similar MREs [51]. Moreover, a circRNA has many miRNA-binding sites. In osteoarthritis therapy, a study showed that circ-33186 inhibits miR-127-5p, thus increasing MMP-13 expression, and contributes to OA pathogenesis [8].

### Other mechanisms of circRNAs in osteoporosis

Zheng et al. [37] demonstrated that circCDK8 represses the osteogenic differentiation of PDLSCs by stimulating autophagy activation through mTOR signaling in a hypoxic environment. Apoptosis can be stimulated in osteoblasts via autophagy, thus accelerating osteoporosis. Beclin‐1 could initiate or inhibit apoptosis depending on which Bcl‐2 family member it interacts with [52], while p53 could induce or inhibit autophagy depending on its localization within the cell [53].

## Several signaling pathways regulated by circRNAs in osteoporosis

**Fig. 4:**
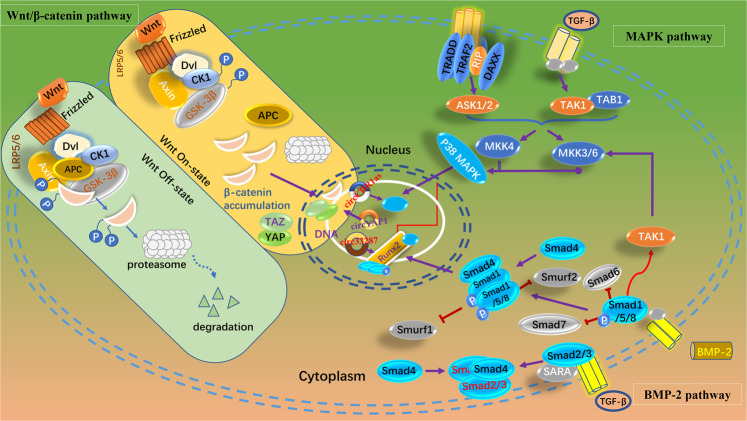
Several signaling pathways regulated by circRNAs in osteoporosis. CircRNAs regulate osteoporosis through several classic signal pathways like the Wnt/β‐catenin signaling pathway, MAPK signaling pathway, and BMP signaling pathway.

Many signaling pathways participate in the regulation of circRNAs in osteoporosis, and several pathways, including the Wnt/β‐catenin signaling pathway, MAPK signaling pathway, and BMP signaling pathway, play vital roles in osteoporosis regulation by circRNAs (Fig. 4).

### Wnt/β‐catenin signaling pathway

The WNT signal transduction cascade mediates various diseases [54]. The Wnt gene was first described in 1982 as an integrated gene in mouse breast tumors [55]. The Wnt/β‐catenin signaling pathway was identified by joint observation in Drosophila and Xenopus and was named the canonical WNT cascade. The Wnt/β‐catenin signaling pathway is a canonical pathway for circYAP1 [56] (Fig. 4).

YAP1 expression is upregulated in MC3T3-E1 cells and promotes osteoblast differentiation during osteoporosis. A TOP/FOP flash assay revealed that circYAP1 elevates YAP1 expression [56] (Fig. 4), and YAP1 was reported to activate the Wnt/β‐catenin signaling pathway [57].

### Mitogen-activated protein kinase (MAPK) signaling pathway

The mitogen‐activated protein kinase (MAPK) signaling pathway has an important impact on osteoblast proliferation and the osteogenic differentiation of mesenchymal stem cells (MSCs). For example, it was found that CDR1as, which is highly expressed in human umbilical cord-derived mesenchymal stem cells (hucMSCs), could regulate the proliferation and differentiation of these cells [35]. In addition, periodontal ligament stem cells could be induced to differentiate into osteoblasts by the circRNA CDR1as via the miR-7/GDF5/SMAD and p38 MAPK signaling pathways [34] (Fig. 4).

### BMP signaling pathway

BMPs belong to the TGF-β superfamily and often play roles in Smad-related pathways. BMP receptors receive messages from BMP ligands and induce the phosphorylation of R‐Smad1/5/8, which facilitates binding with co‐Smad4 and induces the transcription of target genes such as Runx2 and Osterix [58].

BMP2‐induced osteogenesis was confirmed by examining the expression of circRNA_33287, which was upregulated in maxillary sinus membrane stem cells [59] (Fig. 4). In this study, the downregulation of circRNA_33287 inhibited several crucial osteogenic biomarkers, such as Runx2, Osterix, and ALP, while the upregulation of circRNA_33287 exerted the opposite, indicating that circRNA_33287 could induce osteogenesis.

## Conclusion

Focusing on promoting osteoblast formation and inhibiting excessive osteoclastic absorption is the main treatment strategy for osteoporosis. Therefore, it is critical when developing treatments for osteoporosis to comprehend the mechanisms of osteoblast differentiation and osteoclast differentiation. The main signs of osteoblast and osteoclast differentiation have been identified, including WNT signaling and the RANK/RANKL pathway, but it is still necessary to uncover the molecular mechanisms of osteoblast and osteoclast differentiation. Regulatory functions of circRNAs have been identified in various biological processes. These studies demonstrate that circRNAs are important regulators of osteoblast and osteoclast differentiation.

Unlike other noncoding RNAs, the association between circRNAs and osteoporosis is still unclear, and more in-depth research is needed to solve key problems, such as identifying new protocols for detecting circRNAs and clarifying the main regulatory mechanisms of circRNAs in osteoporosis. In addition, the lack of effective therapeutic clinical application is an urgent problem.

## Data Availability

All data generated or analyzed during this study are included in this published article.
